# The Tetrodotoxin Receptor of Voltage-Gated Sodium Channels—Perspectives from Interactions with μ-Conotoxins

**DOI:** 10.3390/md8072153

**Published:** 2010-07-13

**Authors:** Robert J. French, Doju Yoshikami, Michael F. Sheets, Baldomero M. Olivera

**Affiliations:** 1 Department of Physiology and Pharmacology, University of Calgary, and Hotchkiss Brain Institute, 3330 Hospital Drive N.W., Calgary, Alberta, T2N 4N1, Canada; 2 Department of Biology; University of Utah; Salt Lake City, UT, USA; E-Mails: yoshikami@bioscience.utah.edu (D.Y.); olivera@biology.utah.edu (B.M.O.); 3 The Nora Eccles Harrison Cardiovascular Research & Training Institute and Department of Internal Medicine, University of Utah, Salt Lake City, UT 84112, USA; E-Mail: sheets@cvrti.utah.edu

**Keywords:** guanidinium toxins, conopeptides, pore block

## Abstract

Neurotoxin receptor site 1, in the outer vestibule of the conducting pore of voltage-gated sodium channels (VGSCs), was first functionally defined by its ability to bind the guanidinium-containing agents, tetrodotoxin (TTX) and saxitoxin (STX). Subsequent studies showed that peptide μ-conotoxins competed for binding at site 1. All of these natural inhibitors block single sodium channels in an all-or-none manner on binding. With the discovery of an increasing variety of μ-conotoxins, and the synthesis of numerous derivatives, observed interactions between the channel and these different ligands have become more complex. Certain μ-conotoxin derivatives block single-channel currents partially, rather than completely, thus enabling the demonstration of interactions between the bound toxin and the channel’s voltage sensor. Most recently, the relatively small μ-conotoxin KIIIA (16 amino acids) and its variants have been shown to bind simultaneously with TTX and exhibit both synergistic and antagonistic interactions with TTX. These interactions raise new pharmacological possibilities and place new constraints on the possible structures of the bound complexes of VGSCs with these toxins.

## 1. Introduction

Tetrodotoxin (TTX) was the first pharmacological agent targeted to voltage-gated sodium channels (VGSCs) that was characterized (see Narahashi [1] for a personal historical account). A variety of pharmacologically active compounds, which affected VGSC function, were subsequently characterized. In order to provide a rational framework for the analysis of these very different compounds, Catterall and co-workers [2–4] classified the known sodium channel ligands according to their sites of action. This has remained an important differentiating scheme in sodium channel pharmacology. Thus, every compound believed to act at the same pharmacological site as tetrodotoxin is referred to as a ligand of neurotoxin receptor site 1. Saxitoxin (STX) and its derivatives are associated with paralytic shellfish poisoning, which occurs during harmful algal blooms, and are among the most important site 1 ligands that were identified after the discovery of TTX [5].

**Figure 1:**
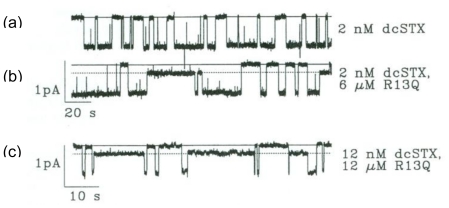
Steady-state recordings from a single, rat skeletal muscle sodium channel enable discrete recognition and analysis of blocking events induced by derivatives of both STX and the conotoxin, μCTX GIIIA. The channel has been modified by batrachotoxin to prevent inactivation. Voltage-dependent activation gating and block by site 1 ligands are retained. (**a**) Typical all-or-none block, resulting from guanidinium toxins, such as TTX and STX, exemplified by the saxitoxin derivative, decarbamoyl-STX (dcSTX); (**b**) After addition of peptide μCTX GIIIA[R13Q], prominent partial blocking events are seen, reducing current by ~70%. The fully blocked/closed level is indicated by the solid line; the partially blocked level produced by R13Q is indicated by the dotted line; (**c**) After increasing the concentrations of both dcSTX and R13Q, the channel is bound most of the time, but in a manner consistent with simple, competitive binding, each fully blocked state (dcSTX-bound), and each partially blocked state (R13Q-bound), is preceded by an unblocked state, which is relatively brief at these high concentrations of the toxins. Adapted from French *et al.*, Neuron, 1996 [18].

From animal venoms, one family of peptides has been identified that consists of site 1 ligands: the μ-conotoxins expressed in the venom ducts of fish-hunting cone snails. It is noteworthy that other venom components, which target different physiological sites on the Na channel, are much more widely distributed among different venoms, but, to date, site 1 compounds have only been reported from cone snail venoms. These peptides are generally regarded as part of the prey-capture cocktail that the snails have evolved, and are considered to be part of the “motor cabal” group of peptide toxins, which act in a coordinated and synergistic fashion to completely inhibit neuromuscular transmission in the envenomated prey [6]. The archetypal μCTX GIIIA was shown to induce all-or-none block of single, skeletal-muscle VGSCs, and to compete for site 1 in binding assays [7,8]. Later, it was found that even the incompletely blocking derivative GIIIA[R13Q] competed for exclusive occupancy of site 1 (see Figure 1).

In passing, we note that, in addition to μ-conotoxins, at least three additional families of peptides from cone snails have been identified that target VGSCs. Members of all three families are gating modifiers: μO-conotoxins, which block channel activation; δ-conotoxins, which block channel inactivation; and ι-conotoxins (or iota-conotoxins), which promote channel activation [6,9]. Thus, μ- and μO-conotoxins are channel antagonists, whereas δ- and ι-conotoxins are channel agonists.

As the only currently known peptide ligands for site 1, the μ-conotoxins have been exploited to identify which amino acids on the Na channel are located around site 1 [10]. Variation in site 1 among Na channel subtypes from different species can, in principle, be investigated through the interactions between the different Na channels and μ-conotoxins or derivatives of μ-conopeptides [7,8,11]. Thus, μ-conopeptides are attractive probes for the exploration of the topology and three-dimensional structure of this pharmacologically important site [12–14]. In addition, the net positive charge on the peptides allows them to participate in numerous long-range electrostatic interactions, which can contribute to binding [15] and to the blocking of ion conduction [16,17]. Electrostatic interactions also modulate gating [18] and drug binding at locations other than site 1, e.g., the amine binding site that makes up part of the local anaesthetic receptor, site 9 [18,19]. This latter interaction may be mediated, at least in part, via interactions with permeant ions [20].

Of particular interest is recent work investigating the interaction between μ-conopeptides and TTX/STX, which reveals that “site 1”, as conventionally defined by pharmacological criteria, is likely a more complex biochemical entity than previously envisioned. TTX is thought to occupy a site within the vestibule of the ion channel near the extracellular end of the channel pore, and, by binding to this site, to occlude the permeation pathway of sodium ions through the pore. The prevailing view is that μ-conopeptides, being larger than tetrodotoxin, occupy a site that spatially overlaps the TTX binding site, but that amino acid residues in the ion channel vestibule further out from the mouth of the pore can contribute to binding of the peptide ligand, even though they do not interact with TTX. In this view, site 1 has two sub-sites: a core that can be occupied by a guanidinium toxin or a μ-conopeptide, and a more peripheral zone that interacts with the peptide, but not TTX or STX.

## 2. Newly Discovered Complexities of Interactions between TTX and μ-Conopeptides

In the sections that follow, we discuss recent work that reveals that the notion of competitive occupancy of the site 1 core, by either a μ-conopeptide or tetrodotoxin molecule, needs to be revisited. In some cases, both ligands can simultaneously bind to a given channel, contradicting the expectation that site 1 ligands are mutually exclusive in binding to site 1. Furthermore, some experimental evidence indicates that even though a μ-conopeptide is bound at site 1 and attenuates channel conductance, it is apparently still possible for TTX to “sneak” past the conopeptide and bind to its site that is presumably deeper in the outer vestibule of the channel to totally occlude the pore.

**Figure 2:**
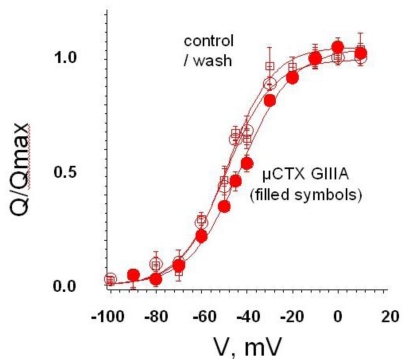
μCTX GIIIA, with its nominal net charge of +6, shifts voltage dependent gating charge movement to the right, which is consistent with the cationic peptide impeding outward movement of the positive charge on the channel’s voltage sensor [21]. This is consistent with shifts in activation of both single channels in lipid bilayers and whole-cell currents [18]. Recording from fused tsA-201 cells expressing rat skeletal muscle VGSCs (rNa_V_1.4) [22].

Recent investigation of potential interactions between TTX and μ-conopeptides has its origins in early observations with single-channel recordings [11,18]. Mutation of the critical arginine residue that, in μCTX GIIIA, is believed to interact directly with the pore, results in a residual Na current that persists when the channel is occupied by the peptide (e.g., GIIIA[R13Q]). The residual current allows the lifetime and function of the toxin-bound channel to be electrophysiologically monitored. Thus, it could be shown that bound toxin influenced the channel’s voltage sensor, a reflection of the proximity of the latter to site 1 [18]. This influence is further illustrated by the direct effect of μCTX GIIIA on gating charge movement (Figure 2). A reversible, positive shift in the plot of gating charge, Q, *vs.* voltage, V, indicates an inhibition of the outward motion of the positive charges on the voltage sensor when the polycationic toxin (nominal net charge, +6) is bound at site 1.

Of possible interest in this regard is the peptide Tx1 from the South American armed spider *Phoneutria nigriventer*, which has been reported to have a binding site that overlaps with that of μ-GIIIA, but not that of TTX [23]. The reported voltage dependence of sodium current block by Tx1 could, in principle, arise either from a direct interaction with the voltage sensor, or from state-dependent binding, but more Fexperiments will be needed to clarify the mechanism.

**Figure 3:**
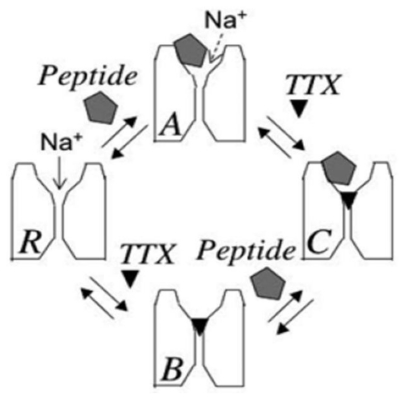
Reaction scheme showing simultaneous binding of TTX and μCTX KIIIA. Reproduced by permission from Zhang *et al.* 2009, Channels [25].

The residual currents of GIIIA derivatives were not blocked by decarbamoyl STX [18] (also see Figure 1), which is consistent with the classical notion that binding of μ-conotoxin and guanidinium toxins to site 1 was mutually exclusive. Residual currents, reminiscent of those observed with GIIIA derivatives and Na_V_1.4, have recently been observed in single channel measurements of the blocking of brain VGSCs by the newly discovered μ-conotoxin KIIIA [24]. Those observations inspired us to see whether: (A) a corresponding residual current could be observed when macroscopic currents were studied, and if so, (B) if the macroscopic current were susceptible to block by TTX [25]. Indeed, for Na_V_1.2 expressed in oocytes that are exposed to saturating concentrations of KIIIA, a residual current (rI_Na_), with an amplitude of 5% of the control I_Na_, was observed. With the mutant KIIIA[K7A], the rI_Na_ was 23% of the control. A key discovery was that the addition of TTX to Na channels, with already bound KIIIA or KIIIA[K7A], produced a complete block, and that the onset of total block was very slow. One possible interpretation was that the peptide dissociated from the channel and was replaced by TTX, thereupon resulting in a complete block of channel conductance. However, several observations established that the peptide did not necessarily dissociate from the channel, and that a ternary peptide TTX Na_V_ complex was formed (Figure 3).

Most notably, there was a dramatic change in the time course of recovery when both TTX and the μ-conopeptide under these conditions were washed out. The rate of recovery was slower than that of following exposure to TTX alone, and faster than that of following exposure to peptide alone. The most straightforward explanation for this result is that both ligands were simultaneously bound to the outer vestibule of the channel, but that TTX was “trapped” at its site by the binding of the peptide, and, as a consequence, TTX could not dissociate with its usual rapid kinetics. However, binding of TTX also affected μ-conopeptide dissociation, because the off-rate for the peptide under these conditions was significantly faster than that which was observed in the absence of TTX. Reciprocally, the rate of dissociation of the peptide limited the off-rates for TTX, and these off-rates (*C*→*A* and *C*→*B*, respectively, Figure 3) differed from those observed for either the peptide (*A*→*R*) or TTX (*B*→*R*) alone. Also, the results suggested that the extremely slow on-rate for TTX (*A*→*C*) was not limited by dissociation of the peptide, but rather, reflected the slow rate of access by TTX to its binding site, when the peptide was bound to the channel.

The rate of conversion from a partially blocked to a totally blocked channel, and the rate of functional recovery, after complete block had occurred and both toxins were washed out, therefore provide a measurable set of parameters that could be monitored using different μ-conopeptides and analogs of μ-conopeptides. One could also compare the effect of replacing TTX by STX, or various analogs of guanidinium toxins [26]. The net result is a coherent picture of interactions between guanidinium toxins and μ-conopeptides (and their analogs) at site 1.

Both the on-rate of the guanidinium toxin in the presence of μ-conopeptide and the off-rate of the toxin in the presence of TTX or STX are dependent on which specific μ-conopeptide (or analog) and which guanidinium toxin is being examined. In some cases, the guanidinium toxin cannot “sneak” past a μ-conopeptide at all. The interaction is determined in part by the charge of the guanidinium toxin and the placement of these charges; these factors influence the effect occupancy that the guanidinium toxin site has on the rate of μ-conopeptide dissociation, and can be rationalized by a charge repulsion model.

## 3. Potential Applications of Double Occupancy of Site 1

The possibility that the guanidinium toxins and certain μ-conopeptides or analogs of μ-conopeptides can simultaneously occupy site 1 raises some interesting pharmacological opportunities. At this time, these are only conceptual constructs, and proof-of-principle for real-world application needs to be worked out. However, since some unusual prospects are raised, especially with regard to modulating the pharmacology of channel block, we present some of the possibilities.

### 3.1. Syntoxins

When co-occupancy by peptide and guanidinium toxin of site 1 occurs, the two partners that occupy the site necessarily interact with each other. Thus, a selective alteration of one of the partners can cause a change in the interaction with the other partner. Thus, a μ-conopeptide that can sit in site 1 simultaneously with TTX is what we regard as a syntoxin of TTX. When the μ-conopeptide is bound, the dissociation rate of TTX is slowed; thus, one can imagine having a series of μ-conopeptide syntoxins resulting in a range of off times for TTX, depending on which μ-conopeptide syntoxin is co-occupying site 1.

### 3.2. Contratoxins

In principle, as the co-occupancy of site 1 by TTX/STX and μ-conopeptides is better understood, it should be possible to design analogs of μ-conopeptides that inhibit TTX, STX, and their analogs from bypassing the peptide to cause complete inhibition of channel conductance. One might imagine a tightly bound μ-conopeptide analog that prevented any guanidinium toxins from reaching their pharmacological site. μ-Conopeptides with such properties could be used as a contratoxin, or antidote, to a red-tide poisoning. Administration of a contratoxin that has a significant residual current could, in principle, partially reverse the more life-threatening effects of the binding of the guanidinium toxins to VGSCs. Thus, by designing a μ-conopeptide derivative with a large residual current and yet very high affinity for site 1, an antidote for red tide poisoning might be produced.

## 4. Recent Results

Since submission of the current manuscript, two papers, which provide additional insight into the complexity of interactions between the μ-conotoxins and derivatives of TTX and STX, have been published online [26,27].
